# Therapeutic potential of L-carnitine in coronary artery disease: a systematic review

**DOI:** 10.1007/s10787-025-02048-7

**Published:** 2026-02-19

**Authors:** Rehab H. Werida, Sherouk M. Okda

**Affiliations:** 1 Department of Clinical Pharmacy and Pharmacy Practice, Faculty of Pharmacy, Damanhour University, Damanhour, 22514 Al Bahaira Egypt; 2 Department of Clinical Pharmacy, Faculty of Pharmacy, Alsalam University, Kafr AL Zayat, 31611 AL Gharbia Egypt

**Keywords:** L-Carnitine, Coronary artery disease, Ischemic heart disease, Antioxidant, Anti-inflammatory

## Abstract

**Background:**

Coronary artery disease (CAD) persists as a major global health burden, contributing significantly to both morbidity and mortality rates worldwide, mostly attributable to atherosclerosis and oxidative stress. L-carnitine (LC), a natural derivative of amino acid, plays a critical role in mitochondrial fatty acid transport and has demonstrated potential antioxidant as well as anti-inflammatory effects.

**Aim:**

This review aims to provide an integrated synthesis that bridges mechanistic evidence (anti-inflammatory, antioxidant) with clinical outcomes (mortality, arrhythmias) for LC supplementation in CAD, while critically appraising inconsistencies across the literature (e.g., heart failure, reinfarction).

**Methods:**

A systematic literature search was conducted in PubMed and Google Scholar databases until July 2025. Studies, including animal studies, case reports, cross-sectional studies, observational studies, retrospective analysis, randomized controlled trials, systematic review and meta-analyses, that investigating the effects of L-carnitine on cardiac function, oxidative stress, inflammation, and mortality in CAD patients were included. Articles that were not within the scope of the study, non-English papers, and those without translations were excluded. A total of 21 studies were identified based on the inclusion criteria.

**Results:**

Across mechanistic endpoints, LC was associated with reductions in inflammatory markers, oxidative stress indices, and cardiac injury biomarkers, with several trials noting improvements in left-ventricular function and lipid profiles. Regarding clinical endpoints, meta-analyses showed reductions in the incidence of all-cause mortality, ventricular arrhythmia, and anginal episodes. In contrast, results were inconsistent regarding heart failure and myocardial reinfarction outcomes.

**Conclusions:**

L-carnitine supplementation may offer cardioprotective benefits in CAD patients; however, given the inconsistent results regarding certain clinical endpoints, further large-scale, long-term randomized trials are required.

**Graphical abstract:**

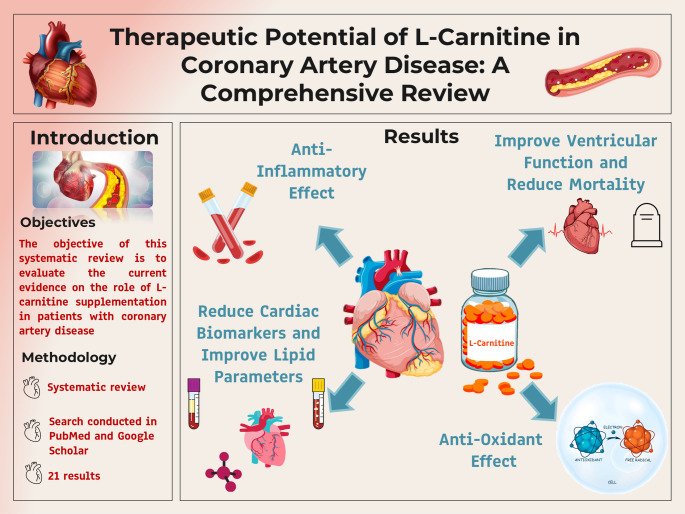

## Introduction

Cardiovascular disease (CVD) is the predominant cause of death worldwide, with a substantial proportion attributable to ischemic heart disease, caused by atherosclerotic coronary artery disease (CAD) (Glovaci et al. 2019; Sarrafzadegan and Mohammadifard 2019). Atherosclerosis is primarily characterized by inflammation, disrupted blood flow, and arterial wall remodeling, substantially influenced by excess reactive oxygen species (ROS) (He and Zuo 2015).

L-carnitine (LC) is a natural compound present in human cells (Johri et al. 2014). This compound is a non-protein amino acid derived biosynthetically from the essential amino acids’ methionine and lysine (Walter et al. 2004). L-carnitine plays a crucial physiological function in lipid metabolism (Lee et al. 2016). L-carnitine facilitates the mitochondrial βeta-oxidation process and energy production by mediating the transport of long-chain fatty acids into the mitochondrial matrix (Ribas et al. 2014). Beyond its metabolic functions, L-carnitine demonstrated antioxidant and anti-inflammatory effects, protecting tissues from oxidative damage induced by reactive oxygen species (Lee et al. 2016; Moeinian et al. 2013). The results of a prior study confirmed the protective benefits of L-carnitine due to suppression of inflammation, oxidative stress, and increased autophagy (Khedr and Werida 2022). During ischemic conditions, myocardial LC levels fall rapidly; supplementation can replenish depleted stores (Wang et al. 2018). This is particularly relevant because the cardiac muscle has limited capacity to synthesize LC (Flanagan et al. 2010)^.^ Additionally, by preventing fatty-acid accumulation and excess lactate production, LC may help enhance myocardial function (Bremer 1983). The antioxidant and scavenging properties of L-carnitine, helped to ameliorate Trazodone’s cardiotoxicity that was caused by an increase in oxidative stress, cardiac enzymes, myocardial inflammation, and autophagy (Khedr et al. 2022). Several experimental and clinical studies have suggested that L-carnitine supplementation may confer cardiovascular benefits in CAD patients, including improved cardiac metabolism, reduced oxidative stress, attenuation of inflammatory responses, and enhanced functional outcomes (Da Silva Guimarães et al. 2017; Dastan et al. 2015; Lee et al. 2014; Lee et al. 2016; Pastoris et al. 1998; Singhai et al. 2017; Tarantini et al. 2006; Xue et al. 2007).

However, previous reviews emphasize common mechanistic pathways such as inflammation and oxidative stress while emerging pathways particularly autophagy-related signaling are under explored. Additionally, most studies also focused on post myocardial infarction injury underexploring L-carnitine effects on procedural injury after percutaneous coronary intervention (PCI). Moreover, mechanistic readouts and clinical endpoints are rarely integrated. To our best knowledge, this is the first systematic review to address these gaps by synthesizing evidence in CAD patients across mechanistic markers such as oxidative stress, inflammatory mediators, autophagy-related markers and post-PCI myocardial-injury biomarkers together with clinical endpoints such as ventricular arrythmia, mortality and angina.

## Methods

### Search strategy

Extensive literature search was performed for articles in PubMed and Google Scholar databases until July 13, 2025. Keywords such as “L-carnitine,” “L-carnitine supplementation,” “coronary artery disease,” and “ischemic heart disease” were used in varied ways, using “and” as a search operator. Supplemental search was performed in cited references.

### Inclusion criteria

This review considered peer-reviewed studies of L-carnitine supplementation in subjects with coronary artery disease across predefined designs, each with a distinct role, including preclinical evidence such as animal studies which provided mechanistic evidence and insights on emerging pathways, as well as clinical evidence such as case reports, cross-sectional studies, observational studies, retrospective analyses, randomized controlled trials, systematic review and meta-analyses which provided primary evidence as well as quantitative analysis of clinical endpoints.

### Exclusion criteria

This review excluded papers that had not fit the scope of the review and those not published in English or for which no English translation was available.

## Results

**Fig. 1:**
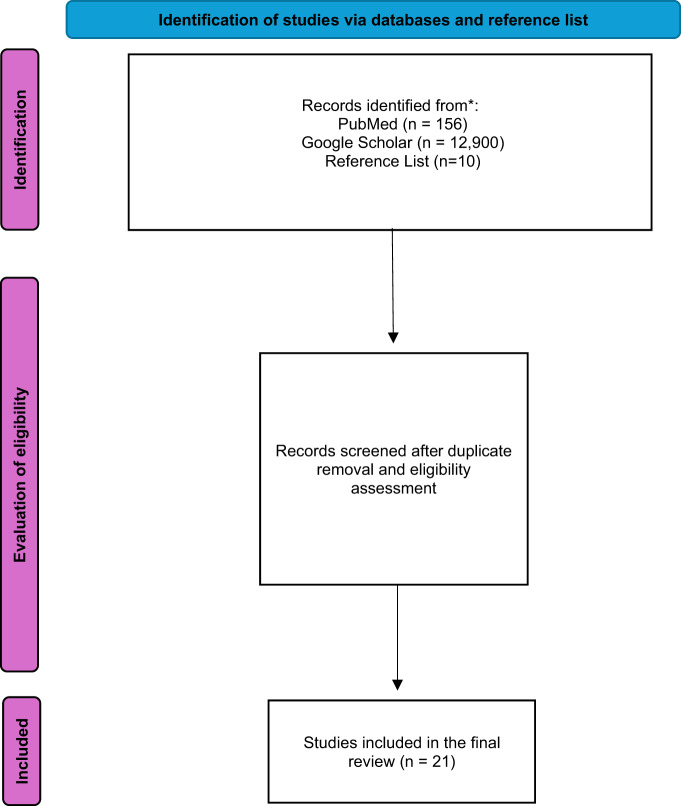
Study flow diagram of the enrolled trials

The search yielded in PubMed 156 results and in google scholar 12,900 results as shown in Fig. 1. The search on the references list yielded 10 results. After meeting inclusion and exclusion criteria and removing duplicates, the search produced 21 results as shown in Table 1.

**Table 1:** Summary of the enrolled trials (n = 21)

References	Study design, sample size, and follow-up	Outcome	Findings
*Randomized controlled trials (RCTs)*			
(Lee et al. 2015)	Single-blind, randomized, parallel, placebo-controlled trial, conducted on 39 coronary artery disease (CAD) patients (placebo, n = 19; L-carnitine (LC), n = 20), followed up for 12 weeks.	Levels of L-carnitine Antioxidant markers: (malondialdehyde and activities of antioxidant enzymes). Inflammatory markers: C-reactive protein (CRP), interleukin-6 (IL-6), and tumor necrosis factor-α (TNF-α).	Following L-carnitine supplementation, inflammatory marker levels were significantly reduced compared to both baseline values and the placebo group. Additionally, these markers demonstrated a strong negative correlation with L-carnitine levels.
(Nachvak et al. 2020)	Double-blind, randomized, placebo-controlled trial, conducted on 75 CAD patients (placebo, n = 37; L-carnitine, n = 38), followed up for 3 months.	Inflammatory markers: high-sensitivity C-reactive protein (hs-CRP), myeloperoxidase (MPO), nitrotyrosine (NT), Total antioxidant capacity (TAC)	After 12 weeks of L-carnitine administration, a significant increase in serum total antioxidant capacity (TAC) and notable reductions in myeloperoxidase (MPO), nitrotyrosine (NT), and high-sensitivity C-reactive protein (hs-CRP) levels were observed compared to the placebo group.
(Lee et al. 2016)	Single-blind, randomized, parallel, placebo-controlled trial, conducted on 47 CAD patients assigned to placebo (n = 24) and to L-carnitine (n = 23), followed up for 12 weeks.	Levels of LC. Lipid profile. Antioxidant enzyme activity: (superoxide dismutase, SOD).	L-carnitine supplementation at a dose of 1000 mg/day resulted in a significant increase in HDL-C and Apo-A1 levels, along with a slight reduction in triglyceride levels. However, no significant changes were observed in other lipid parameters or in antioxidant enzyme activity among patients with coronary artery disease.
(Xue et al. 2007)	Open label, Single blind randomized placebo-controlled trial, conducted on 96 Non-ST Elevation Myocardial Infarction (NSTEMI) patients (control, n = 48; L-carnitine, n = 48) and followed up for 30 days.	Cardiac markers: creatine kinase-MB and troponin-I were assessed immediately prior to and 8-, 12- and 24-h post-PCI Composite endpoint: recurrent ischemia, myocardial infarction and mortality during hospitalization and within 30 days post discharge.	L-carnitine treatment independently predicted a reduction in creatine kinase-MB and troponin-I levels.
(Furat et al. 2018)	Prospective randomized trial, conducted on 60 patients undergoing coronary artery bypass grafting, followed up 48 h post-surgery.	Cardiac output, cardiac index, and right and left Ventricular stroke work Cardiac markers: lactate dehydrogenase (LDH), creatinine phosphokinase (CPK), CK-MB, Troponin-I.	Patients receiving L-carnitine exhibited significant improvements in cardiac output, cardiac index, and both right and left ventricular stroke work. Furthermore, the L-carnitine group demonstrated a marked reduction in Troponin-T levels.
(Singhai et al. 2017)	Prospective randomized trial, conducted on 100 CAD patients (placebo, n = 50, L carnitine, n = 50), followed up for 12 weeks.	CRP levels.	CRP levels were significantly reduced from baseline following 12 weeks of L-carnitine supplementation in the treatment group.
(Dastan et al. 2015)	Randomized controlled trial, conducted on 134 patients scheduled for CABG, who were randomly assigned to either a control group or an L-carnitine group and followed up for 24 h postoperatively.	Cardiac markers: CK-MB and troponin T.	No significant difference in CK-MB or troponin T levels was observed between L-carnitine and control groups.
(Lee et al. 2014)	Single blind, randomized, parallel, placebo-controlled trial, conducted on 39 CAD patients, followed up for 12 weeks.	Levels of LC. Oxidative stress markers: MDA, CAT, SOD, and GSH-Px.	LC supplementation markedly diminished MDA levels and substantially elevated the functional activity of antioxidant enzymes such as CAT, SOD, and GSH-Px. The concentration of LC exhibited positive correlation with the activity of antioxidant enzymes.
(Da Silva Guimarães et al. 2017)	Randomized controlled trial conducted on 28 patients undergoing CABG, (19 in L-carnitine group, 9 in placebo group), followed up for 180 days post-CABG.	Echocardiographic parameters: Left ventricular ejection fraction (LVEF) Left ventricular systolic/diastolic diameters (LVFSD/LVFDD).	Although no significant intergroup differences were noted in reverse remodeling parameters, the L-carnitine group demonstrated a significant improvement in left ventricular ejection fraction (LVEF) along with substantial reductions in left ventricular end-systolic diameter (LVESD) and end-diastolic diameter (LVEDD).
(Tarantini et al. 2006)	Randomized, double-blind, multicenter, placebo-controlled trial, conducted on 2330 patients with acute myocardial infarction, followed up for 6 months.	Primary endpoint: composite of death and heart failure at 6 months. Secondary endpoint: 5-day mortality.	At the 6-month follow-up, no significant difference in the primary endpoint was observed between the L-carnitine and placebo groups. However, a reduction in mortality was noted in the L-carnitine group on day 5.
(Singh et al. 1996)	Randomized, double-blind placebo controlled trial in patients with suspected acute myocardial infarction, (placebo n = 50, L-carnitine n = 51), followed up for 28 days.	Infarct size assessment: Cardiac enzymes: CK and CK-MB. Electrocardiographic (ECG) changes: QRS-score. Metabolic markers: 1. AST and LDH. 2. Lipid peroxides. Cardiac events: 1. Angina pectoris. 2. Heart failure severity. 3. Left ventricular enlargement. 4. Arrhythmias, Cardiac deaths.	L-carnitine supplementation led to reduction in infarct size, heart failure severity, arrhythmia, and total cardiac events compared to placebo.
*Preclinical studies*			
(Khedr et al. 2022)	Animal study, conducted on 32 male albino rats (n = 8 per group). (Control, Trazodone, L-carnitine, L-carnitine + Trazodone), followed up for 4 weeks.	Cardiac markers: (AST & CK-MB) and serum cardiac troponin T(cTnI). Oxidative stress biomarkers: (malondialdehyde; MDA, total thiol, and catalase (CAT) activity). Autophagy-related genes: (ATG-5 and Beclin-1), P62 and TNF-α.	Daily supplementation of L-carnitine (200 mg/kg) entirely mitigated the trazodone-induced elevation of cardiac enzymes, autophagy, and myocardial inflammatory responses.
(Wang et al. 2020)	Animal study, conducted on 32 Wister rats divided into 4 groups: a control group on normal diet (n = 8), a normal diet plus LC group (n = 8), an atherosclerosis group (n = 8), and an atherosclerosis + LC group (n = 8), followed up for 16 weeks.	Lipid parameters: total cholesterol (TC), triglycerides (TG), low-density lipoprotein (LDL), very low-density lipoprotein (VLDL), and high-density lipoprotein (HDL). Oxidative stress markers: SOD, Glutathione Peroxidase (GSH-Px), MDA levels. Inflammatory markers: TNF-α, IL-1β, CRP, Inducible nitric oxide synthase (iNOS), and adiponectin.	Acetyl-L-carnitine markedly improved lipid parameters by lowering total cholesterol, triglycerides, LDL, and VLDL levels while increasing HDL. It also enhanced antioxidant defenses (elevated SOD and GSH-Px, reduced MDA) and attenuated inflammation by decreasing markers such as TNF-α, IL-1β, iNOS, and CRP; however, it had no significant impact on adiponectin levels.
*Review articles (systematic reviews and meta-analysis)*			
(DiNicolantonio et al. 2013)	A systematic review and meta-analysis of 13 controlled trials, (n = 3629 patients), range of follow-up (3 weeks–12 months).	The effects of L-carnitine on mortality, ventricular arrhythmias (VAs), angina, heart failure, and myocardial reinfarction.	Compared to the placebo, L-carnitine supplementation was significantly associated with reduced rates of all-cause mortality, ventricular arrhythmias, and angina onset; however, it did not lower the incidence of heart failure or recurrent myocardial infarction.
(Lango et al. 2001)	Review article summarizing the results of animal and clinical studies of L carnitine effects on myocardial metabolism and function.	Animal studies: 1. ATP concentration in myocardium. 2. Ventricular contractility. 3. Coronary blood flow. 4. Arrhythmia incidence. Clinical studies: Ischemic heart disease: 1. Mortality reduction. 2. Lactate uptake by myocardium. Heart failure: 1. Ejection fraction improvement. 2. Cardiopulmonary Bypass: 3. ATP preservation in atrial muscle Stroke volume post-surgery.	In patients with acute myocardial ischemia LC supplementation resulted in ↓ Mortality, ↓ circulatory failure, ↑ lactate extraction In patients in heart failure L-carnitine administration resulted in ↑ Ejection fraction In patients with cardiopulmonary bypass L-carnitine administration resulted in ↑ ATP stores, better mitochondrial function when added to cardioplegia.
(Shang et al. 2014)	Meta-analysis of five controlled trials, (n = 3108), range of follow up (1–12 months).	The effects of L-carnitine on all-cause mortality and cardiovascular morbidity in patients with acute myocardial infarction (AMI).	No significant changes in mortality or cardiovascular outcomes were observed across different dosage levels of L-carnitine.
(Elantary and Othman 2024)	Review article about the role of L-carnitine in Cardiovascular Health that included systematic reviews, meta-analyses, randomized controlled trials, and observational studies. Sample size (47–1625), and range of follow up (12 weeks-12 months).	Primary outcomes: 1. Left ventricular ejection fraction 2. Ventricular arrhythmias 3. Mortality post-AMI 4. Walking distance in peripheral vascular disease (PVD) patients 5. Oxidative stress and lipid profiles in CAD patients Secondary outcomes: 1. Mitochondrial function and energy metabolism 2. Anti-inflammatory and antioxidant effects	L-carnitine reduced mortality and arrhythmias post-AMI, improved walking distance in PVD patients, and lowered oxidative stress in CAD patients. However, concerns included potential pro-atherogenic effects resulting from the formation of trimethylamine N-oxide (TMAO) by gut microbiota metabolism.
(DiNicolantonio et al. 2014)	Review article summarizing clinical trials and meta-analyses about L-carnitine effects on AMI patients, Sample size (18–2330), and range of follow up (5 days-12 months).	Primary outcomes: 1. Reduction in infarct size (measured by cardiac enzymes as CK-MB, troponin-I). 2. Decreased ventricular arrhythmias (VAs) and mortality. 3. Attenuation of left ventricular (LV) remodeling. Secondary outcomes: 1. Improved glucose oxidation and mitochondrial efficiency in ischemic tissue. 2. Reduced angina incidence and oxidative stress.	L-carnitine reduced mortality, arrhythmias, angina, infarct size, and left ventricular remodeling, however had no consistent benefit for the incidence of heart failure or reinfarction.
(Serban et al. 2016)	A systematic review and meta-analysis of randomized controlled trials (n = 7) summarizing the impact of L-carnitine on plasma lipoprotein(a) concentrations, range of follow up (1 week-24 weeks).	Primary outcome: Plasma Lipoprotein (a) concentrations. Secondary Outcomes: Total cholesterol, LDL-C, HDL-C, and triglycerides.	L-carnitine, particularly when administered orally, led to a significant reduction in lipoprotein (a) and total cholesterol levels. However, it had no noticeable impact on HDL or triglycerides, and intravenous L-carnitine showed no significant effect.
(Wang et al. 2018)	Review article summarizing preclinical and clinical studies about L-Carnitine and heart disease, range of follow-up (3 months-1 year).	Primary outcomes: 1. Reduction in ventricular dysfunction, ischemia–reperfusion injury, cardiac arrhythmias, and toxic myocardial injury. 2. Improvement in metabolic parameters (e.g., fatty acid oxidation, ATP production). Secondary outcomes: 1. Attenuation of hypertension, hyperlipidemia, diabetic complications, and oxidative stress.	L-carnitine enhanced left ventricular function, reduced infarct size and mortality following myocardial infarction, protected against ischemia–reperfusion and toxic myocardial damage.
(Ferrari et al. 2004)	Review article summarizing preclinical and clinical studies about the effects of L-Carnitine and Propionyl-L-carnitine on cardiovascular Diseases, range of follow-up (1 month to 12 months).	Primary outcomes: 1. Reduction in ventricular dysfunction, ischemia–reperfusion injury, and arrhythmias. 2. Improved exercise tolerance and metabolic parameters. Secondary outcomes: 1. Anti-ischemic and antianginal effects. 2. Attenuation of left ventricular remodeling post-AMI.	L-carnitine and propionyl-L-carnitine enhanced left ventricular function, exercise capacity, ischemia, and arrhythmias in cardiac patients.

### Anti-inflammatory effects

A study by lee et al., reported that after LC administration, patients in the L-carnitine group demonstrated significantly lower levels of inflammatory markers including C-reactive protein (CRP), interleukin-6 (IL-6), and tumor necrosis factor-alpha (TNF-α) compared to both baseline values and the placebo group. Furthermore, post-supplementation analyses revealed a negative correlation between TNF-α concentrations and L-carnitine levels (Lee et al. 2015).

In the study conducted by Nachvak et al., L-carnitine supplementation significantly increased serum total antioxidant capacity (TAC) and significantly reduced levels of myeloperoxidase (MPO), nitrotyrosine (NT), and high-sensitivity C-reactive protein (hs-CRP) compared to the placebo group. These results suggest that L-carnitine effectively attenuated inflammatory and oxidative stress markers. Furthermore, the L-carnitine group exhibited greater reductions in anthropometric parameters, including lean body mass (LBM) and body fat mass (BFM), relative to the placebo group (Nachvak et al. 2020). An animal study reported that L-carnitine supplementation markedly reduced the concentrations of TNF-α, IL-1b, and CRP in Wister rats. Although there was a slight decrease in adiponectin (APN) levels, the difference did not reach statistical significance (Wang et al. 2020).

A study conducted on 100 CAD patients demonstrated a significant reduction in CRP levels in the L-carnitine group after 12 weeks of supplementation compared to baseline (Singhai et al. 2017) as shown in Table 1.

### Effect on oxidative stress

In an animal study conducted by Wang et al., L-carnitine administration led to a significant elevation in Superoxide Dismutase (SOD) and Glutathione Peroxidase (GSH-Px) levels in both serum and cardiac tissue, accompanied by a marked reduction in malondialdehyde (MDA) levels. These findings suggest that L-carnitine effectively attenuated oxidative stress in atherosclerotic rat models (Wang et al. 2020).

Similarly, a clinical study involving patients with coronary artery disease demonstrated that 12 weeks of L-carnitine supplementation resulted in reduced malondialdehyde levels, increased circulating L-carnitine concentrations, and enhanced activities of catalase and glutathione peroxidase enzymes compared to the placebo group. Moreover, L-carnitine levels showed a significant positive correlation with the activity of these antioxidant enzymes (Lee et al. 2014).

### Effect on cardiac markers

One animal study demonstrated that administration of LC mitigated the cardiotoxic effects of trazodone (TRZ)-induced cardiotoxicity by normalizing levels of creatine kinase-MB (CK-MB), aspartate transaminase (AST), and cardiac troponin I (cTnI) compared to TRZ only group. Moreover, LC alleviated the elevated expression of autophagy protein P62 in heart tissue observed in the TRZ group. Additionally, co-administration of LC with TRZ significantly increased total thiol content and catalase activity while reducing malondialdehyde (MDA) levels relative to TRZ alone (Khedr et al. 2022).

Furthermore, administration of L-carnitine substantially altered the effects of TRZ and markedly reduced the expression of autophagy-related genes such as Beclin1, Autophagy Related Gene 5 (ATG5), and the inflammatory mediator TNF-α in comparison to the TRZ treated group (Khedr et al. 2022).

Another study reported that creatine kinase-MB levels were lower in the L-carnitine group compared to the control group at both 12- and 24-h post-treatment. Additionally, Troponin-I levels were significantly reduced in the L-carnitine group relative to the control group 8 h after percutaneous coronary intervention (PCI) (Xue et al. 2007).

In accordance with a previous study, a clinical study conducted on Coronary Artery Bypass Grafting (CABG) patients demonstrated a pronounced reduction in cardiac markers such as Troponin-T, lactate dehydrogenase (LDH) and CK-MB in the L-carnitine-supplemented group (Furat et al. 2018).

Similarly, a clinical study in patients with suspected acute myocardial infarction found that L-carnitine treatment led to a significant reduction in infarct size, indicated by both cardiac marker levels (creatine kinase and CK-MB) and electrocardiogram (ECG) QRS-scores. Additionally, patients administered L-carnitine had a lower incidence of severe heart failure, left ventricular hypertrophy, arrhythmias, and total cardiac events, including mortality and nonfatal myocardial infarctions, compared to those receiving placebo (Singh et al. 1996).

One study reported that L-carnitine supplementation decreased lipoprotein (a) levels in cardiac patients (Serban et al. 2016).

Contrary to previous findings, a study by Dastan et al. demonstrated that L-carnitine administration had no significant impact on CK-MB or cardiac troponin T levels relative to the control group, either before or after CABG surgery (Dastan et al. 2015) as shown in Table 1.

### Effect on mortality

A pervious clinical study conducted on Anterior ST Segment Elevation Myocardial Infarction patients reported a significant reduction in the 5-day mortality in the L-carnitine treatment group as a secondary endpoint. However, mortality rates between day 7 and day 180 were comparable between the L-carnitine and control groups (Tarantini et al. 2006).

According to a systematic review and meta-analysis, L-carnitine supplementation significantly reduced all-cause mortality by 27%, the incidence of ventricular arrhythmias by 65%, and the occurrence of angina by 40% compared to placebo. However, no significant effect was observed on the rates of heart failure or myocardial reinfarction (DiNicolantonio et al. 2013).

Similarly, another review reported that L-carnitine supplementation in patients with acute myocardial infarction resulted in reduced rates of both mortality and circulatory failure. In addition, propionyl-L-carnitine was found to improve lactate clearance by 43% and increase stroke volume by 8% in patients with ischemia. Furthermore, high-dose L-carnitine significantly lowered the incidence of ventricular arrhythmias following myocardial infarction (Lango et al. 2001).

Consistent with previous results, several review articles and meta-analyses reported beneficial effects of L-carnitine supplementation on mortality and cardiovascular outcomes in cardiac patients (AlMosawi 2021; DiNicolantonio et al. 2014; Elantary and Othman 2024; Wang et al. 2018).

There was a discrepancy between the previous results and the results of a meta-analysis, which demonstrated no significant effect of L-carnitine on all-cause mortality or major cardiac events, including heart failure, unstable angina, and myocardial reinfarction. (Shang et al. 2014).

### Effect on echocardiography

Furat et al. reported that L-carnitine supplementation in patients undergoing CABG significantly enhanced cardiac output, cardiac index, as well as left and right ventricular stroke work index both immediately following cardiopulmonary bypass and during the first postoperative hour (Furat et al. 2018).

Similarly, another study also conducted on CABG patients indicated that the L-carnitine group exhibited a significant increase in left ventricular ejection fraction (LVEF) 180 days post-surgery compared to baseline. Furthermore, the Left ventricular systolic diameter (LVFSD) also decreased significantly in the L-carnitine group. However, no significant changes were observed in left ventricular diastolic diameter (LVFDD) in either group. Overall, although the L-carnitine group exhibited significant within-group improvements in systolic function, intergroup comparisons revealed no statistically significant differences (Da Silva Guimarães et al. 2017).

### Effect on lipid parameters

One study demonstrated that following 12 weeks of LC supplementation, participants in the LC group showed significantly increased LC concentrations and enhanced Superoxide dismutase (SOD) activity compared to the placebo group. In terms of lipid profile, the LC group had markedly higher levels of high-density lipoprotein cholesterol (HDL-C) and apolipoprotein A1 (Apo-A1) than those receiving the placebo. Additionally, LC levels were negatively associated with triglyceride (TG) levels. Negative correlation was also observed between SOD activity and lipid markers such as total cholesterol (TC) and apolipoprotein B (Apo-B) after LC supplementation (Lee et al. 2016).

One animal study reported that following the administration of acetyl L-carnitine to Wistar rats, the serum levels of TC, TG, Low-Density Lipoprotein (LDL), and Very Low-Density Lipoprotein (VLDL) in the L-carnitine group were significantly reduced compared to the atherosclerosis group, while the level of HDL was markedly elevated (Wang et al. 2020).

## Discussion

### Anti-inflammatory effects

Atherosclerosis and coronary artery disease are now widely recognized as chronic inflammatory conditions rather than merely the result of lipid accumulation within the vasculature (Granger and Kvietys 2015). Reactive oxygen species are considered key triggers that amplify the inflammatory process (Hansson 2005).

Among the main inflammatory mediators, CRP plays a crucial role in atherogenesis by promoting complement activation, enhancing macrophage lipid uptake, stimulating pro-inflammatory cytokine release, inducing monocyte tissue factor expression, impairing endothelial function, and inhibiting nitric oxide production, further aggravating vascular injury (Shrivastava et al. 2015).

In this review, L-carnitine supplementation consistently demonstrated anti-inflammatory effects, as evidenced by significant reductions in inflammatory markers including CRP, TNF-α, and IL-6 across multiple clinical and animal studies. Suggesting a valuable role for LC in modulating inflammatory pathways in CAD.

### Effect on oxidative stress

LC has demonstrated significant antioxidant effects. Recent in vivo and in vitro investigations have shown that LC can mitigate oxidative damage in models of cardiovascular disease by diminishing lipid peroxidation, scavenging reactive oxygen species such as hydrogen peroxide and superoxide, chelating metal ions involved in oxidative stress (Brodericksq et al. 1992; Eysiaks et al. 1988; Gülçin 2006; Kolodziejczyk et al. 2011; Mingorance et al. 2011), and enhancing the endogenous antioxidant capacity, including the upregulation of CAT, SOD, and GSH-Px activities (Augustyniak and Skrzydlewska 2010; Binienda and Ali 2001; Li et al. 2012; Miguel-Carrasco et al. 2010).

Structurally, L-carnitine’s carbonyl group at the α-carbon can stabilize free radicals and shield plasma components from the harmful effects of reactive oxygen species and reactive nitrogen species. (Ribas et al. 2014; Scioli et al. 2019). Given that reactive oxygen species (ROS) may trigger inflammation by promoting pro-inflammatory cytokines and activating the NF-κB pathway, LC’s antioxidant action indirectly reinforces its anti-inflammatory effects. (Setia and Sanyal 2012). Research has demonstrated that antioxidants, such as LC, can suppress the NF-κB activation pathway, hence diminishing oxidative and inflammatory stress (Conner and Grisham 1996; Kurutas et al. 2005).

Furthermore, one of the mechanisms by which L-Carnitine supplementation may reduce oxidative stress is through the preservation of mitochondrial function by enhancing catalase and superoxide, which are antioxidant enzymes (Le Borgne et al. 2017). Evidence indicates that the concentration of LC in ischemic heart disease diminishes in cardiac muscle (Wang et al. 2018). A decrease in antioxidant enzymes can expedite the progression of atherosclerosis (Ou et al. 2017). That’s why Supplementation with LC may help restore this balance and slow the progression of atherosclerotic cardiovascular disease.

Consistent with these mechanisms, according to the included studies L-carnitine supplementation was associated with lower MDA levels indicating reduced lipid peroxidation and higher SOD and GSH-Px activities which underscores its antioxidant effect in patients with CAD.

### Effect on cardiac ischemia and cardiac outcomes

L-carnitine’s theoretical benefit in improving clinical outcomes, particularly after ischemic events and cardiac surgeries, is well-established. In the setting of myocardial ischemia, the buildup of long-chain acyl-CoA within mitochondria interferes with ATP transport, thereby compromising cellular metabolism and viability (Furat et al. 2018).

The primary rationale for the therapeutic benefits of L-carnitine is its ability to restore normal oxidative metabolism and replenish energy stores in the myocardium. It facilitates the transport of potentially harmful fatty acids into mitochondria within ischemic heart tissue, thereby supporting cellular energy production (Ferrari et al. 2004). It also reduces the intramitochondrial ratio of acetyl-CoA to free CoA, which in turn stimulates pyruvate dehydrogenase activity and promotes pyruvate oxidation. (Ferrari et al. 2004). These metabolic shifts help preserve high-energy phosphate reserves, minimize ischemic damage, and enhance ventricular recovery upon reperfusion (Ferrari et al. 2004). Furthermore, LC prevents the accumulation of long-chain acyl-CoA, thereby reducing the risk of life-threatening ventricular arrhythmias during ischemia (Cui et al. 2003).

Moreover, L-carnitine mitigates post-ischemic myocardial injury primarily by counteracting the toxic impact of increased free fatty acid concentration and promoting more efficient carbohydrates metabolism (Ferrari et al. 2004). Furthermore, L-carnitine enhances the restoration of oxidatively damaged membrane phospholipids and attenuates apoptosis triggered by ischemia, thereby limiting subsequent left ventricular remodeling (Cui et al. 2003).

Carnitine is the primary determinant of fatty acid turnover in mitochondria. Carnitine depletion occurs rapidly during prolonged ischemia (Carvajal and Moreno-Sánchez 2003). Exogenous administration may provide significant mechanical benefits through various mechanisms: reduction of infarct size in the risk area (Ura 1990), enhancement of functional recovery in post-ischemic myocardium (Tarantini et al. 2006), and lastly, limitation of adverse post-myocardial infarction left ventricular remodeling (Tarantini et al. 2006).

The antioxidant properties of L-carnitine are associated with its function in mitochondrial fatty acid transport, which prevents the accumulation of free fatty acids in the cytosol. The observed reduction in both systolic and diastolic left ventricular diameters, along with improved left ventricular ejection fraction in the LC supplemented group, suggests a potential contribution of CABG to reverse remodeling. This effect is likely mediated by enhanced energy production in surviving cardiomyocytes. (Da Silva Guimarães et al. 2017). Pastoris et al. demonstrated that L-carnitine supplementation led to elevated myocardial creatine content and enhanced cardiac performance in patients undergoing CABG (Pastoris et al. 1998).

According to Oyanagi et al., l-carnitine reduces cardiac cell death, mediates oxidative stress, inhibits the synthesis of fatty acid esters during ischemia events, and aids in the removal of toxins from inside the mitochondria (Oyanagi et al. 2011). Serum levels of CK-MB, AST, and cTnI were dramatically reduced after receiving 200 mg/kg l-carnitine for four weeks, which significantly reduced TRZ cardiotoxicity (Khedr et al. 2022). Clinical research showed that supplementing L-carnitine can improve myocardial fat metabolism because it increases the need for free fatty acids and their metabolites, which improves cardiac performance (DiNicolantonio et al. 2013; Malone et al. 2006).

Additionally, L-carnitine’s therapeutic potential in managing cardiotoxicity has been studied in a variety of rat models (Elkomy et al. 2020; Jaswal et al. 2011). Xanthine oxidase, NAD(P)H oxidases, and cytochrome P450 catalyze enzymatic processes in the heart that produce reactive oxygen species during normal mitochondrial cellular functions during oxidative phosphorylation (Keleş et al. 2014). Structural changes such as cardiomyopathy, cardiac hypertrophy, interstitial cardiac fibrosis, endothelial dysfunction, and contractile protein failure can result from elevated oxidative stress in cardiac tissue (Emran et al. 2021). However, the improvement of the histological markers suggests that the heart was successfully protected by the L-carnitine treatment.

Consonance with these mechanistic pathways, the results of this review indicate favorable cardiac outcomes with L-carnitine supplementation, including reduced infarct size as well as lower incidence of heart failure, ventricular arrhythmia, total cardiac events, and all-cause mortality.

There were discrepancies in the effects of L-carnitine on cardiac biomarkers, heart failure, reinfarction, and all-cause mortality. These may be due to heterogeneity across studies and meta-analyses. Principal effect modifiers are speculated to be differences in patient populations (peri-operative CABG vs PCI vs medically managed acute myocardial infarction) as well as resultant ischemic burden (Furat et al. 2018; Xue et al. 2007). Moreover formulation (L-carnitine vs propionyl-/acetyl-L-carnitine), dose, route, timing relative to ischemia/reperfusion, and treatment duration of L carnitine (short peri-procedural courses vs chronic supplementation) may contribute to these differences (Da Silva Guimarães et al. 2017; Ferrari et al. 2004; Furat et al. 2018; Xue et al. 2007). Background care (concomitant use of statins, angiotensin converting enzyme inhibitors /angiotensin receptor blockers, beta-blockers and dual antiplatelet therapy) may also play a role in this discrepancy. Additionally, study design/quality (small single-center trials vs larger multicenter RCTs) can influence observed effects (Lee et al. 2016; Tarantini et al. 2006). Collectively, these factors provide a biologically and methodologically plausible basis for divergent findings across outcomes.

Collectively, despite the noted discrepancies, these findings underscore LC’s multifaceted role in protecting the myocardium during ischemic events and surgical interventions, reinforcing its potential as a valuable adjunct in the management of CAD.

## Conclusion

L-carnitine supplementation exhibits multiple beneficial effects in patients with coronary artery disease, including reduction in inflammation, oxidative stress, lipid abnormalities, and cardiac injury markers. Clinical trials and meta-analyses indicate improvements in ventricular function and reductions in mortality, arrhythmias, and angina. Despite some conflicting findings, particularly regarding heart failure and reinfarction, L-carnitine remains a potentially valuable adjunct therapy. While the overall evidence is promising, to move from promise to practice, future multicenter, long-term RCTs, standardizing the dosage and formulation of L-carnitine should be a top priority for future large-scale trials. They should also seek to identify patient subgroups that may benefit the most, such as those with low baseline carnitine levels or undertaking certain procedures like PCI/CABG, evaluating safety and interactions with guideline-directed therapies. Such trials will derive the greatest benefit of L carnitine supplementation in clinical practice.

## Data Availability

The review data will be shared upon request from the corresponding author.

## Declarations

### Human or animal participants

Not required for reviews because they do not involve collecting new data from human participants.
